# Supplementary material for: Insecticide resistance and its intensity in urban Anopheles arabiensis in Kisumu City, Western Kenya: Implications for malaria control in urban areas
Source: PLoS One. 2024 Nov 13;19(11):e0303921. doi: 10.1371/journal.pone.0303921 (PMC11560014; doi:10.1371/journal.pone.0303921)
Supplement: S1 Table — (DOCX) [file pone.0303921.s001.docx]

**Insecticide resistance and its intensity in urban *Anopheles arabiensis* in Kisumu City, Western Kenya: *Implications for malaria control in urban areas***

Maxwell G. Machani^1,3*^, Irene Nzioki^1,^ Shirley A. Onyango^2^, Brenda Onyango^1^, John Githure^3^, Harrysone Atieli^3^, Chloe Wang^5^, Ming-Chieh Lee^5^, Andrew K. Githeko^1^, Yaw A. Afrane^4^, Eric Ochomo^1^ and Guiyun Yan^5^

**S1 Table** : Types of pesticides and their use as reported by residents in urban , peri-urban and rural zones, western Kenya.

| Zones | Pesticide Class (% in use) | Trade name | Active ingredients | Purpose |
| --- | --- | --- | --- | --- |
| Urban | Pyrethroid ( 39 %) | Biothrin | Deltamethrin25g/L | Veterinary |
|  |  | Vectocid 5% EC | Deltamethrin 50gm/ltr |  |
|  |  | pentagon 50EC | Lambda-cyhalothrin 50g/l |  |
|  |  | Halothrin | Lambda- cyhalothrin 2.5% |  |
|  |  | Ectomin 100EC | Cypermethrin high-cis 100g/L |  |
|  |  | Duduthrin | Lambda-cyhalothrin 17.5 g/lt. | Veterinary/ public health (bedbug control) |
|  |  | Vendex 50EC | Lambdacyhalothrin 50g/L | Crops pest control |
|  |  | Bestox 100 EC | Alphacypermethrin 10% |  |
|  |  | Jackpot 50 EC | Lambda-Cyhalothrin | Public health(bedbug control) |
|  |  | Grenade EC | lambda-cyhalothrin 5% |  |
|  | Organophosphate (11.5%) | Gladiator | Chlorpyrifos 480g/L | Public health(bedbug control) |
|  |  | Oshothion 50EC | Malathion 50% w/v | Veterinary |
|  |  | Diazonon | O,O-Diethyl O phosphorothioate | Crops/public health (bedbug control) |
|  | Carbamates (3.8%) | sevin Dudu dust | Carbaryl 7.5% | Veterinary |
|  | Neonicotinoids(11.5%) | Acetak | Acetamiprid 200g/L | Veterinary/ public health (bedbug control) |
|  |  | bedlam | Acetamiprid 200g/L | Public health(bedbug control) |
|  |  | Bamako 700wg | Imidacloprid 30.5% |  |
|  | Pyrethroid+organophosphate( 11.5%) | Rocket 44EC | Profenofos 40% + Cypermethrin 4% | Veterinary/ public health (bedbug control) |
|  |  | profile 440 EC | Profenos 40% +Cypermethrin4% |  |
|  |  | Cyperdip 55 EC | Cypemethrin 50g/L & chloropyrofos 500G/L. | Veterinary |
|  | Pyrethroids+Neonicotinoids(3.8%) | Thunder | Imidacloprid (100g/l) + Beta-cyfluthrin (45g/l). | Crops pest control |
|  | pyrethroid+ synergist (3.8%) | Vampire | Pyrethrins 3.00%, Piperonyl butoxide, Technical 30.00% | Public health(bedbug control) |
|  | Pyrethroid+ synergist+organophosphate (3.8%) | vectoclor plus EC | Cypermethrin 15g/l, chloropyrofos 25g/l, piperonyl butoxide 15g/l, citronella 1g/l | Veterinary |
|  | Amidine group (11.5%) | Norotraz | Amitraz 12.5% | Veterinary |
|  |  | Actraz 12.5 EC | Amitraz 12.5% |  |
|  |  | Triatix | Amitraz 12.5% |  |
| Periurban | Pyrethroids ( 36.4%) | Halothrin 2.5 EC | Lambda- cyhalothrin 2.5% | Veterinary |
|  |  | Albaz 10 EC | Alphacypemethrine 10% | Crops pest control |
|  |  | Delete EC | Deltamethrin 50gm/L | Veterinary |
|  |  | Ambush EC | Permethrin 500g/L | Crops pest control |
|  | Organophosophate ( 9%) | Gladiator | Chlorpyrifos 480g/L | Public health(bedbug control) |
|  | Carbamates (9%) | Sevin Dudu dust | Carbaryl 7.5% | Veterinary/ public health (bedbug control) |
|  | Neonicotinoid (18.2%) | Bedlam | Acetamiprid 200g/L | Public health(bedbug control) |
|  |  | Acetak | Acetamiprid 200g/L | Veterinary |
|  | Amidine group(18.2%) | Triatix | Amitraz 12.5% EC | Veterinary |
|  |  | Mostraz 12.5EC | Amitraz 12.5% EC |  |
|  | Ryanoid (9%) | Cover | Rynaxypyr 0.4% G | Public health(bedbug control) |
| Rural | Pyrethroid (37%) | Delete EC | Deltamethrin 50gm/L | veterinary |
|  |  | Biothrin | Deltamethrin25g/L |  |
|  |  | Vectocid 5% EC | Deltamethrin 50gm/L |  |
|  |  | pentagon 50EC | Lambda-cyhalothrin 50g/l |  |
|  |  | Ectomin 100EC | Cypermethrin high-cis 100g/L |  |
|  |  | Umeme top 50EC | Lambda-cyhalothrin 50g/L |  |
|  |  | Duduthrin 1.75EC | Lambda-cyhalothrin 17.5 g/L | Veterinary/ public health (bedbug control) |
|  | Organophosphate (11%) | Danadim | Dimethoate 40% | Crops pest control |
|  |  | Gladiator | Chlorpyrifos 480g/l | Public health(bedbug control) |
|  | Carbamates (5.3%) | Sevin dudu dust | Carbaryl 7.5% | Veterinary/ public health (bedbug control) |
|  | Pyrethroid+organophosphate(16%) | Dabotik 522.5 Ec | Chlorpyrifos 475g/l + cypermethrin 47.5g/l. | Veterinary |
|  |  | Sidai ultra dip | Cypemethrin 50g/L & chloropyrofos 500G/L. |  |
|  |  | Umeme Duodip 55* Ec | Chlorpyrifos 500g/l and Cypermethrin 50g/l |  |
|  | Pyrethroid+Neonicotinoids (5.3%) | Epic 130 sc | Imidacloprid 100g/L +Lambda-Cyhalothrin 30g/L | Crops pest control |
|  | Amidine group ( 26.3%) | Actraz 12.5%Ec | Amitraz 12.5% | Veterinary |
|  |  | Tixfix | Amitraz 12.5% |  |
|  |  | Norotraz | Amitraz 12.5% |  |
|  |  | Triatix | Amitraz 12.5 % |  |
|  |  | Farmtraz | Amitraz 12.5% |  |
